# Supplementary material for: Patterns of Intron Gain and Loss in Fungi
Source: PLoS Biol. 2004 Nov 30;2(12):e422. doi: 10.1371/journal.pbio.0020422 (PMC532390; doi:10.1371/journal.pbio.0020422)
Supplement: Table S1 — Also available at http://genes.mit.edu/NielsenEtAl/. (4.3 MB ZIP). [file pbio.0020422.st001.zip › NielsenEtAl/html/802.html]

AN7755.1.NCU01954.1.MG02960.1.FG08901.1


```
 CLUSTAL W (1.82) Multiple Sequence Alignments - Introns Inserted


Sequence 1: MG02960.1	335 aa
Sequence 2: FG08901.1	328 aa
Sequence 3: NCU01954.1	405 aa
Sequence 4: AN7755.1	332 aa
Alignment Length: 405 aa
Number Identitical Residues: 146 aa
Alignment Score (without introns) 7485


MG02960.1 	--------------------MDAPAAPVAVFKKRGAKAKANIRKRAATPPPAD----SDS
NCU01954.1	MADTEQPPTAPTAPEPTTATPTAPIAPVAIFKKRGAKGKANLRKRPASPPPAAKDSDDDS
FG08901.1 	MADS----------------TTEPQAPVAVFKKRGAKGKANIRKRPATPPPAD----SDD
AN7755.1  	---------------------MADTAPQVSFKKRSVK-KTNFRKKPESPPPDA----DSD
          	                         ** . ****..* *:*:**:. :***      ...

MG02960.1 	SDYSSSEDEA-GQRIKRRRKDK-SGNVTTSTKTSAAADAD--------LGATVFKADRSV
NCU01954.1	SDFESSEDEATGQRIKRRKKNHHSAAVMASSRDHNASTNSGVTKEEQIRSNTIYEADRDA
FG08901.1 	SDYSSSEDES-GQRVKRRKK---TATVTASSKDLTSGKKD--------FSATIYSADRSV
AN7755.1  	SSFTSSDDEE-GHRIKRRRK---NAAVSASSTSNTRRTTT---------SDEPATAGAAV
          	*.: **:**  *:*:***:*   .. * :*:                  .     *.  .

MG02960.1 	PI---TDSNDATKRSNWFDA-EDMDSLSTKNLMGTTRSAQQ---------------QEPP
NCU01954.1	ALRLDATKQDATKGSNWFDEDNEKEDLSVGNLLGRTRTMMKTKKKVGGEDDAVQNSEREP
FG08901.1 	PI---TSTNDATKHSDWYE--EDKNELSAKNLLGSTRGPTK---------------DSQP
AN7755.1  	PL---TASNDATKHSNWYD-----DELNEKNLLGTTRARPAATG------------ADAP
          	.:   : .:**** *:*::     :.*.  **:* **    :.                *

MG02960.1 	DGKYKGLANKPSFIQKNPDAPSRTVGPVKAPTNIRTITITDFAPDVCKD0YKQTGFCGFG
NCU01954.1	DGTYRGLANQASYIQKNPNAPNRKVGPIKAPSNIRTITITDMAPDVCKD2YKQTGFCGFG
FG08901.1 	DGTYKGLANQTSFIQKNPDAPQRAKGPVKAATNIRTITVMDFKPDICKD~YKKTGHCGFG
AN7755.1  	DGTYKGAANYQSFIQKNPNAPAKTFGPIKAPTNVRTVTFMDYAPDVCKD~YKLTGYCGFG
          	**.*:* **  *:*****:** :  **:**.:*:**:*. *  **:*** ** **.****

MG02960.1 	DNCKYLHAREDYKAGWQLDKEWETVTKGKKNLGGTVVASRDKSNA-----AAAEEDDDEE
NCU01954.1	DNCKFLHAREDYAHGWQLDKEWENVTKGKKNLQGTVVASADRRNKPKNTAEEEEDADAAE
FG08901.1 	DSCIFLHDRTDVKQGWQLDKEWEEVTKGKKNLGGTIIGSSSRDKK-----EQAPEDE-AE
AN7755.1  	DSCKFSHMREDYKQGWELDRDWEVSTKGK-NLGGKVVSQRG---------GQAGEDEDDE
          	*.* : * * *   **:**::**  **** ** *.::.. .             : :  *

MG02960.1 	DALLESIPFACIICKEPYK-QPIKTRCGHYFCEPCALKRYRKDPTCAACGSGTNGVFNGA
NCU01954.1	EALLENIPFACIICRGPYSNSPVVTRCGHYFCEACALKRYRKDPSCAACGSGTNGVFNAA
FG08901.1 	IAMLEKIPFACIICEGPYR-EPIVTRCGHYFCEPCALKRYRKDPTCASCGAGTNGVFNSA
AN7755.1  	EEQLENIPFACIICKKPYQ-NPIVTKCGHYFCESCALQRYRKNPSCAACGAGTGGVFNVA
          	   **.********. **  .*: *:*******.***:****:*:**:**:**.**** *

MG02960.1 	RQLQKLLDKKRERAAKRRQAAIEAGEEVSDEEEEE-----------
NCU01954.1	KRLAKLLEKKKARAERLRREARERGEEVSEEEDEGEDEGEGAEGSD
FG08901.1 	KKLKKLLEKKKEREEKKK----KAEEEAADEE--------------
AN7755.1  	KKLNGLLEKKRERARQRREQAIANGEEVSSEEDEDEDEEQNSS---
          	::*  **:**: *  : :  :    **.:.**.. ... ..:.
```
